# STARC-SUD – Adaptation of a Transdiagnostic Intervention for Refugees With Substance Use Disorders

**DOI:** 10.32872/cpe.5329

**Published:** 2021-11-23

**Authors:** Annett Lotzin, Jutta Lindert, Theresa Koch, Alexandra Liedl, Ingo Schäfer

**Affiliations:** 1 Department of Psychiatry and Psychotherapy, University Medical Center Hamburg-Eppendorf, University of Hamburg, Hamburg, Germany; 2 Centre for Interdisciplinary Addiction Research, University of Hamburg, Hamburg, Germany; 3 University of Applied Sciences, Emden / Leer, Emden, Germany; 4 Women`s Research Center, Brandeis University, Waltham, MA, USA; 5 Refugio München, Munich, Germany; University of Lausanne, Lausanne, Switzerland

**Keywords:** emotion regulation, affect regulation, substance use disorders, addiction, refugees, group treatment, cultural adaption, formative research

## Abstract

**Background:**

Refugees often suffer from multiple mental health problems, which transdiagnostic interventions can address. STARC (Skills-Training of Affect Regulation – A Culture-sensitive Approach) is a culturally sensitive transdiagnostic group intervention that has been developed for refugees to improve affect regulation. In refugees with substance use disorders (SUD), the consideration of SUD-specific elements might improve the acceptance and effectiveness of such an intervention. We aimed to adapt the STARC program for refugees with SUD in a culturally sensitive way.

**Method:**

The conceptual framework of Heim and Kohrt (2019) was used to culturally sensitively adapt the STARC program to the needs of Syrian refugees with SUD. The results of five focus group discussions with refugees on cultural concepts of SUD and their treatment informed the adaption. An expert group suggested adaptions and decided by consensus on their implementation. Two pilot groups were conducted with the adapted STARC-SUD program. Interviews with the therapists of these pilot groups informed further adaption.

**Results:**

The concepts related to SUD identified in focus groups and therapists’ interviews that differed from Western concepts were integrated into the STARC intervention.

**Discussion:**

Further studies should assess the acceptance and effectiveness of the culturally sensitive STARC-SUD program for refugees with SUD.

## Highlights

- The study offers insight into the adaptation process of a culturally sensitive group intervention.
- We report the adaptation of a group intervention for refugees with substance use.
- Cultural concepts of Syrian refugees related to substance use are considered in the adaptation.

The rising global burden of forced migration is one of the most pressing public health issues (UNHCR, 2019). Forced migration is related to many stressors that increase the risk for SUD, including loss of loved ones, different types of abuse, family separation, social and economic inequality, and discrimination in the host country (Horyniak et al., 2016). In refugees, substance use disorders (SUD) have received increasing awareness (Horyniak et al., 2016), with a prevalence rate of hazardous or harmful alcohol use ranging from 4% to 7% in community settings (Horyniak et al., 2016). The availability of substances and the often higher acceptance of substance use in the host country (e.g., drinking alcohol in public) might additionally increase the risk of SUD (Priebe et al., 2016).

While there is a need for SUD health care for refugees, this need often is not met (Welbel et al., 2013). Several barriers to access services exist. Lack of knowledge about the mental health care system in the host country prevents access (Posselt et al., 2017). In addition, refugees are often required to attend multiple psychosocial services before entering SUD treatment, risking disengagement. Interpreters are unavailable, or if available, the health insurance does not cover the costs (Jaeger et al., 2019). Additional barriers to accessing SUD health services concern different concepts of suffering and SUD-related stigma (Penka et al., 2012). The lack of knowledge and skills in cultural sensitivity in professionals further contributes to the SUD health care gap among refugees.

The culturally sensitive adaption of the existing Western evidence-based interventions seems central to reduce barriers to mental health care in refugees. The adaption of the language, culture, and context of an intervention to be compatible with the user’s cultural patterns, meanings, and values (Bernal et al., 2009) may enhance its acceptability and effectiveness (Hall et al., 2016). Indeed, evidence has accumulated that cultural adaptations enhance the efficacy of treatments based on Western psychotherapeutic approaches in populations with other cultural backgrounds (Anik et al., 2021; Chowdhary et al., 2014).

As refugees often suffer from multiple mental disorders, the need for evidence-based transdiagnostic treatments has received increasing attention (Martin et al., 2018). Transdiagnostic interventions address mechanisms underlying common mental disorders. Such interventions may be preferable to disorder-specific interventions, as therapists can apply them to a group of refugees with heterogeneous symptoms. Group therapy with people who have survived the same experience seem to be more effective than individual therapeutic approaches (Kira et al., 2012).

A few transdiagnostic treatment approaches have been developed for non-Western cultures. Problem Management Plus (PM+; Dawson et al., 2015) is a five-session low-intensity intervention developed for low and middle-income countries targeting persistent distress and mild symptoms of depression and anxiety (Dawson et al., 2015). PM+ was effective in reducing psychological distress (e.g., Bryant et al., 2017), but no research examined its effects on SUD. “Common Elements Treatment Approach” (CETA) is another brief intervention for common mental health disorders developed for low-resource settings (Murray et al., 2014). CETA effectively reduced hazardous alcohol use in an at-risk sample for interpersonal violence in Zambia (Murray et al., 2020). Culturally sensitive evidence-based interventions for refugees in the middle- or high-income countries are needed to target SUD and other mental disorders in refugees.

## The STARC Intervention

A culturally sensitive group intervention developed for refugees in the Western middle- or high-income countries is STARC (Skills-Training of Affect Regulation – A Culture-sensitive Approach; Koch & Liedl, 2019). STARC is a 14-session culture-sensitive transdiagnostic intervention to improve affect regulation in refugees. The intervention is based on Western skills-based elements from Skills Training in Affective and Interpersonal Regulation therapy (STAIR; Cloitre & Schmidt, 2015), the Dialectic Behavioral Therapy (DBT; Bohus et al., 2011), and the Culturally Adapted Cognitive Behavioral Therapy (CA-CBT; Hinton et al., 2011). The authors developed the STARC program according to guidelines for developing culturally sensitive interventions (Bernal & Sáez-Santiago, 2006). The manual includes culturally-sensitive metaphors and expressions and uses easy language. A pilot study in Afghan refugees indicated preliminary evidence that the intervention reduces difficulties in emotion regulation, general distress, and post-traumatic stress disorder symptoms (Koch et al., 2020).

Difficulties in regulating emotions play a key role in SUD (Aldao et al., 2010). Improving emotion regulation via culturally sensitive interventions such as STARC seems essential to reduce substance use and relapse in individuals with SUD. Such interventions need to address managing emotions effectively to regulate craving when the risk of substance abuse is high. Previous research showed that individuals with SUD benefited from tailored emotion regulation interventions that considered their specific needs, e.g., coping with craving beliefs (Choopan et al., 2016).

While emotion regulation strategies are a centerpiece of the STARC intervention, it does not focus on the interrelations between emotion regulation and substance use. Adapting the STARC intervention for the specific needs of refugees with SUD might further enhance its acceptance and effectiveness in this vulnerable group. Therefore, the aim of this study was to adapt the STARC program for Syrian refugees with SUD.

## Method

The adaption of the STARC program was conducted in preparation of a randomized controlled trial of the STARC-SUD program in refugees with substance use problems (Schäfer et al., 2020), which is part of a research network on the prevention and treatment of substance use disorders in refugees (PREPARE, Prevention, and Treatment of Substance Use Disorders in Refugees; BMBF 01EF1805A). The Ethics Committee of the Medical Council of Hamburg approved this study (PV7123).

### Intervention

The STARC program (Koch & Liedl, 2019) was developed in a participatory approach with refugees. STARC is a weekly group program conducted with six to eight refugees of the same gender and an interpreter if required. It consists of fourteen 90-min sessions. The program contains four modules: 1) Introduction and training of emotional perception; 2) Training of specific emotion regulation strategies; 3) Dealing with specific emotions, and 4) Rehearsal and closure.

Module 1 aims at improving emotional awareness. Emotions and their functions are discussed, and the interrelations between feelings, thoughts, and body reactions are explained. Personal warning signals for different emotional intensities are also introduced. In Module 2, emotion regulation strategies are conveyed, including cognitive approaches, body-based strategies, and strategies to cope with intense feelings. In Module 3, coping with specific emotions, such as anger or fear, is discussed. In Module 4, the group reviews the learned skills and celebrates program completion (for a more detailed description, see Koch & Liedl, 2019).

### Procedure of Adaption

In the current study, we focused on Syrian refugees as they represent one of the largest refugee groups in Germany. Due to restricted resources, we shortened the program to ten sessions. The sessions were reviewed with the authors of STARC, sessions with overlapping content were merged. The shortened STARC program was extended with SUD-specific elements while keeping the basic concept of the program. The STARC sessions were adapted by referring to substance use as a dysfunctional emotion regulation strategy throughout the sessions. In addition, we integrated elements used in SUD group treatment, such as discussions about the risk and protective factors of SUD and the short- and long-term consequences of substance use (Körkel & Schindler, 2003; Lindenmeyer, 2016).

In accordance with Heim and Kohrt (2019), cultural concepts of substance abuse were collected as a first step of the cultural adaption process. Five focus groups with three to nine refugees were conducted to assess their core assumptions, beliefs, and concepts of SUD. The focus group discussions were based on a published interview guideline and followed standard procedures for reporting qualitative studies (Lindert et al., 2021). The focus groups included 19 purposively recruited male adult Syrian refugees. They were aged 20 to 50 years and lived in Germany in metropolitan, urban, or rural areas. A native-speaking professional translator and one facilitator conducted the focus groups. The facilitator was a female PhD student in Psychology with a background in Ethnology. Inductive content analysis (Mayring, 2014) was applied to analyze the transcribed data and extract common themes.

The results of the focus groups with refugees yielded culture-specific information about core assumptions, beliefs, and concepts related to SUD and its treatment with refugees. The results of the focus groups were published in a separate paper (Lindert et al., 2021). Based on the results of the focus groups, three experts proposed adaptions in a standardized adaption sheet. The first expert (second author) was a researcher in the field of migration research; the second expert (first author) was a mental health professional and expert in the field of traumatic stress and psychotherapy research; the third expert was a mental health professional from Afghanistan working with refugees with a flight history. In a consensus meeting, the three experts commented on the suggestions of each other and then discussed and decided on the adaptions. In case of disagreement, the suggestion was discussed together until an agreement between the discussants was reached.

A STARC-SUD prototype was created and then piloted in two groups with Syrian refugees with SUD. The pilot groups were conducted by trained therapists in routine SUD care facilities. The therapists had a German background. All content was translated simultaneously during the sessions. After completion of the program, the therapists were invited to an unstructured interview to provide feedback on their experience with the program. The interviews were conducted by a clinical psychologist experienced in the conduction of group therapies. The interviewer noted the key points in the adaption sheet during the interview. These interviews informed further adaption of the program that were documented and consented by the same expert group in a second consensus meeting.

All adaptations are described in Supplement 1. In accordance with the procedure of Heim et al. (2021), this issue, and Heim and Knaevelsrud (2021), this issue, a standardized template was used to document the adaptations (see Supplement 2). This template includes the following sections: i) target group; ii) formative research methods; iii) cultural concepts of distress (i.e., idioms of distress, explanatory models); iv) target intervention; v) deep structure adaptations (i.e., specific and unspecific elements and in-session techniques); and vi) surface adaptations (i.e., mode of delivery, materials).

## Results

### Cultural Adaption of STARC-SUD

The adapted elements of the STARC program are documented in Supplement 2, the content of the different sessions of the adapted program is described in Supplement 3.

#### 1. Unspecific Elements

The results of the focus group discussions and therapists’ interviews indicated that some refugees were unfamiliar with the Western concept of psychotherapy which suggests that individuals solve mental health problems by themselves (rather than within the family) by consulting a mental health professional. In contrast to this approach, some refugees found it more appropriate to solve mental health problems collectively within the family system. Hence, we included psychoeducation about the concept of Western psychotherapy in the introductory session. Furthermore, the therapists stressed that the approach to talk about mental health problems in a group with other patients needed to be introduced. Therefore, we included psychoeducation about the group setting as a common intervention approach in Western cultures to support and learn from each other in the introductory session.

#### 2. SUD-Specific Elements

Not all refugees shared the concept of SUD as a treatable mental disorder. Consequently, we added information on the Western concept of addiction as a recognized treatable mental disorder and the availability of professional addiction services to the STARC manual. Most refugees stressed that rules and norms differed between the host and home country; the greater availability of substances was perceived as contributing to SUD. The greater societal acceptance of substance use was frequently mentioned as another reason for SUD. Thus, we incorporated information about the substances commonly used in the host and home country, as well as their availability and acceptance in the STARC-SUD program.

Refugees and therapists reported refugee-specific risk factors for SUD, e.g., traumatic experiences in the home countries or during flight, worries about family members that remained in the home country, and not feeling accepted by the host country. Refugee-specific risk factors for SUD were therefore included in the STARC-SUD program. In addition to these refugee-specific risk factors, refugees mentioned culture-specific protective factors for not developing SUD, such as societal and family norms, and social support. These factors were incorporated into the manual.

#### 3. Other Specific Elements

The therapists reported that some of the male refugees hesitated to play a group dynamic game with a ball of wool to get familiar with other group members in the introductory session. These male refugees perceived the game as more appropriate for women. Hence, we changed the manual instruction recommending to be sensitive to gender-based preferences regarding group games.

Some refugees participating in the pilot groups reported being unfamiliar with the relaxation exercises introduced in the program (breathing exercise and Progressive Muscle Relaxation) to regulate tension or intense feelings. Rather, they preferred more active strategies (e.g., physical exercises and singing). We adapted the program to instruct the therapists to offer both relaxation exercises and alternative active strategies.

According to the therapists’ feedback obtained in the interviews, some participants preferred religious statements of encouragement as a strategy to regulate emotions, while others preferred non-religious statements, as they were non-religious or persecuted for religious reasons. Therefore, it was more strongly emphasized in the manual to be mindful in proposing religious rituals, e.g., reading the Koran or Bible, or talking to God or Allah.

#### 4. Treatment Delivery

Some refugees with a high level of education found that the easy language used in the STARC manual appeared unfamiliar to them. Therefore, we added an instruction to the manual that therapists could adapt the complexity of the language according to the language skills and education of the participants.

The therapists reported that the translator needed to have read the manual before the session to translate the content correctly. In addition, therapists emphasized the need of having sufficient time to ensure that all participants correctly understood the translation of the session content, e.g., by asking comprehension questions and providing additional information as needed. A briefing of the translators on the translation procedure before the session might also be helpful. Therefore, we underlined these aspects more strongly in the introductory part of the program.

## Discussion

Based on the focus group discussions with Syrian refugees on cultural concepts of SUD and its treatment, we integrated elements relevant for the treatment of SUD in a culturally sensitive way into the STARC program. After piloting the first version of the STARC-SUD prototype, we further adapted the program based on interviews with the therapists that conducted two STARC-SUD pilot groups.

### Unspecific Elements

We found that some of the refugees were unfamiliar with the Western concept to solve mental health problems with a mental health specialist. This finding is in line with the results of previous research showing that the Western concept of psychotherapy, i.e., to consult a mental health professional to talk about mental health problems, may be unfamiliar to people from non-Western cultures (Gopalkrishnan, 2018). Earlier research also revealed that provision of knowledge about (Western) mental health services and how to access them may increase trust in refugees (Duden et al., 2020; Sandhu et al., 2013). Furthermore, we found that the group setting (vs. individual setting) used for the STARC program needed to be introduced in more detail.

Psychoeducation about the Western concept of (psycho-)therapy as a common approach in German healthcare to cope with mental health problems seems important. This may include discussing the approach to solve problems individually in a professional setting with a health care specialist as an alternative or complementing strategy to collectivistic approaches to enhance understanding, acceptance, and adherence to the program.

### SUD-Specific Elements

In the focus groups that were conducted prior to the cultural adaptation, refugees outlined several SUD-specific aspects as essential to be incorporated in a culturally sensitive intervention (Lindert et al., 2021). These included different concepts and norms for addiction, as well as for substance use, their availability, and acceptance. The finding that some of the refugees were unfamiliar with Western concepts of addiction as a recognized and treatable mental disorder is in line with the results of earlier qualitiatve research among Afghan populations showing that the concepts of mental disorders, such as depression (Alemi et al., 2016) and posttraumatic stress disorder (Yaser et al., 2016), differed from those reported by Western populations. The acceptance of interventions addressing SUD in refugees might be improved by introducing the Western concept of addiction as a recognized treatable mental disorder, and by discussing differences and similarities with other concepts of addiction. Psychoeducation about commonly used substances, their availability and acceptance in the host and home countries might also increase acceptance and adherence to the intervention. Furthermore, our results indicated that refugee-specific risk and protective factors for SUD needed to be considered to provide a relevant model of the development of SUD, e.g., traumatic experiences or worries about family members.

### Other Specific Elements

Gender-specific preferences for dynamic group games needed to be considered in the STARC-SUD program. Previous studies with refugees also reported gender-specific preferences for group therapy content that were related to gender-specific socialization experiences (Kira et al., 2012). These results speak to the importance of conducting gender-separated therapy groups.

We also found that the type of exercises to regulate emotions needed to be chosen culturally sensitively. A study by Somasundaram (2010) indicated that relaxation techniques might be an effective component in treating mental disorders in refugees if they include techniques known and used in the respective culture.

The sensitive use of religious content in the program was another important finding of our study. While some refugees perceived religion as a source of strength, others experienced it as a source of threat. These results indicate the need to consider religious content carefully in mental health interventions for refugees. However, in refugees that perceive religion as a source of strength, religious content in a culturally sensitive intervention might be particularly helpful, as religious believes are an integral part of ones’ own understanding of the world in many non-Western cultures (Machleidt, 2019). Consistent with this assumption, relaxation techniques (Somasundaram, 2010) and therapeutic interventions (Hasanović, 2017) including religious content have been perceived as helpful among refuges in previous research.

### Treatment Delivery

While the easy language used in the program seemed essential to improve the comprehensiveness of the program content for non-native speakers, it became clear that easy language could appear artificial for high-educated refugees, indicating the need for individual adaption of the used language to the participants of the respective intervention.

We also found that the translations improved if the translators read the program sessions beforehand. These findings are consistent with a previous qualitative study by Duden et al. (2020), which reported that patients and mental health providers were concerned that not everything said had been translated correctly. The quality of the translation could be increased by having interpreters that familiarize themselves with the session content in advance. Our results also indicated that enough time-related resources are needed during the session to ensure that all refugees understood the translated content correctly.

Overall, this study identified a number of necessary adaptions of a therapeutic intervention, developed within Western cultures, to the needs of individuals from other cultural backgrounds. Attention should be payed to the clarification of the underlying concepts. For refugees, it might be an unfamiliar concept that speaking about one's problems in groups is appropriate, and learning from others might have healing effects. Moreover, if such therapies include skills-based approaches, there is a need to consider their appropriateness from a gender and culture-sensitive perspective.

Our results indicate implications concerning offering support for SUD in refugees. When adapting Western therapeutic approaches to the needs of refugees with SUD, Western concepts of mental disorders underlying the intervention should be discussed, such as the concept of addiction as a recognized and treatable mental disorder. In addition, the different societal norms for substance use, the types of substances, and their availability and acceptance in the host and home countries should be addressed.

### Limitations

There are limitations concerning the methodology of our cultural adaptation. The program was culturally adapted by integrating non-Western metaphors, opinions from non-Western cultures about diseases and healing, and easy-to-understand language. Nevertheless, it seems impossible to make psychotherapy a culture-free concept, as it is rooted in the Western culture. The database used for our adaption is limited by only considering male refugees. Future studies need to examine the appropriateness of the program for female refugees. Another limitation is that we did not assess sociodemographic characteristics except age to guarantee confidentiality for the study participants.

### Conclusion

According to the results obtained from focus groups (Lindert et al., 2021) and the therapists’ interviews, we adapted several elements in a culturally sensitive way. Although the original version of the STARC manual had already been developed culturally sensitively (Koch & Liedl, 2019), further potentially beneficial adaptations could be made from the sources included in the present study. This suggests that qualitative research such as focus groups should be used to inform cultural adaptions of existing interventions to consider the specific needs of a target group, such as refugees with SUD. Further studies might evaluate whether the cultural and SUD-specific adaptions increase the STARC-SUD intervention's acceptance and effectiveness.

## Supplementary Materials

The Supplementary Materials contain the following items (for access see Index of Supplementary Materials below):

- Supplement 1 describes the adaptation steps of the STARC-SUD intervention.
- Supplement 2 summarizes the results of the focus group discussions with refugees and the interviews with therapists, as well as the adaptations of the STARC-SUD intervention decided by consensus.
- Supplement 3 provides an overview of the adapted sessions of the STARC-SUD intervention.

10.23668/psycharchives.5185 Supplement 1 Supplementary materials to "STARC-SUD – Adaptation of a transdiagnostic intervention for refugees with substance use disorders"

LotzinA.
LindertJ.
KochT.
LiedlA.
SchäferI.
 (2021). Supplementary materials to "STARC-SUD – Adaptation of a transdiagnostic intervention for refugees with substance use disorders"
[Additional information]. PsychOpen. 10.23668/psycharchives.5185
PMC967083236405673
