# Supplementary material for: STARC-SUD – Adaptation of a Transdiagnostic Intervention for Refugees With Substance Use Disorders
Source: Clin Psychol Eur. 2021 Nov 23;3(Spec Issue):e5329. doi: 10.32872/cpe.5329 (PMC9670832; doi:10.32872/cpe.5329)
Supplement: Supplement 1 [file cpe-03-5329-s01.pdf]

Annett Lotzin, Jutta Lindert, Theresa Koch, Alexandra Liedl, & Ingo Schäfer:
STARC-SUD – Adaptation of a transdiagnostic intervention for refugees with substance use disorders. https://doi.org/10.32872/cpe.5329

This article is part of the Special Issue "Cultural Adaptation in Clinical Psychology", published in Clinical Psychology in Europe.

Supplement 1.

Adaption Steps of the STARC-SUD Intervention

Steps	Description
1	Conduction of focus group discussion with refugees to obtain knowledge about concepts of SUD and healing
2	Compilation of a STARC-SUD prototype, informed by the focus groups
3	Piloting of a STARC-SUD prototype in two pilot groups
4	Interviews with STARC-SUD therapists about their experiences with STARC-SUD
5	Expert discussions and consensus of the adaptions
6	Further adaption and finalization

Notes. SUD = Substance Use Disorder. STARC = Skills-Training of Affect Regulation – A Culture-sensitive Approach.

Supplement 2

Adaptation Monitoring Sheet of The Skills-Training of Affect Regulation – A Culture-sensitive Approach (STARC) Intervention

1. Project overview

**Target group and intervention**			
**Category**	**Description**	**Results**	**Source**
Target group	Description of elements that are potentially relevant for cultural adaptation, e.g., language, religion, migration status, age	Adult Afghan and Syrian refugees in Germany with hazardous substance or substance use disorders.	Focus groups
Intervention	STARC-SUD („Skills Training in Affect Regulation – a Culture-sensitive Approach") for refugees with hazardous substance use or substance use disorder	STARC-SUD is a culture-sensitive transdiagnostic cognitive-behavioral group therapy for refugees with hazardous substance use or SUD. The 10-session skills-based intervention targets affect regulation and coping with SUD. Participants are enrolled in groups of four to eight participants homogenous in gender and ethnic background.	STARC-SUD Manual

2. Adaptation: Overview

**Cultural concepts of distress and specific interventions**				
**Category**	**Sub-category**	**Description**	**Results**	**Source**<br>E.g., Literature review (cite the reference), qualitative interviews, focus groups, etc.
Cultural concepts of distress	Core beliefs about human suffering	General assumptions about human suffering and healing	• Suffering reflects problems in the community and society. [a]	Five focus groups with 19 participants from metropolitan, urban, rural areas
Cultural concepts of distress	Concepts about substance use	Explanatory models, aetiological assumptions	• Substance abuse is caused by societal rules of consumption. Substance abuse increases if societies allow substance use. Substances are used more often in Germany because their use is tolerated.[b]<br>• Substances are used more as they are available in the host country. Substances were less used in Syria as they were difficult to obtain. Acceptance of substance use, particularly alcohol, is lower than in Germany, and therefore substance use is lower. [c]<br>• Societal and family norms in the home countries can be protective against substance use, e.g., thinking that the family at home would not like it or would be proud to stop drug use. [d]<br>• Family control is protective: In home countries, family controls if family members take substances. [e]<br>• Moslem religion can be protective as it prohibits alcohol use. [f]<br>• Social support is protective: Social support helps to feel better and to reduce the need to escape from negative feelings. [g]<br>• Substances are used to escape from negative memories, feelings, and traumatic experiences in the home country, during flight or in Germany like loneliness, boredom, lack of social connection, loss of family members, and worries about family members in the home country. [h]<br>• Not being accepted or integrated into the host society causes negative emotions that lead to substance use. [i]<br>• The wish to belong to others and to be integrated into the host country leads to substance use. [j]<br>• During the war, the substance use of tobacco and hashish was seen as resistance towards the Islamic State (IS); therefore, the use of these substances increased. [k]	Focus groups
	Culturally salient symptoms	Symptom patterns	• Marihuana, alcohol, and prescription drugs are the most popular and most often used substances in Syria. [l]	Focus groups
	Disorder-specific assumptions / beliefs	Negative (and positive) beliefs about symptoms/disorders	• Substance abusers are not accepted and stigmatized in society and family, as they behave against the norms. [m]<br>• Substances can be a medicine to help to escape from negative memories and feelings and war-related burdens. [n]	Focus groups
	Idioms of distress	Socially acceptable terms for expressing distress	• Mental health problems should be solved within the family. [o]<br>• People with mental disorders are stigmatized ("being crazy"). [p]	Focus groups

**Cultural concepts of distress and specific interventions**				
**Category**	**Sub-category**	**Description**	**Results**	**Source**<br>E.g., Literature review (cite the reference), qualitative interviews, focus groups, etc.
	Unspecific factors (e.g., empathy, empathic listening)	Elements that are universal to the therapy experience and reflective of the approach used by the therapist to engage a patient or implement the therapy	• Group cohesion and group support: Group setting might be unusual for refugees and needs to be addressed as a common approach in Germany. [w]	Discussion with STARC therapists
	In-session techniques e.g., goal-setting, role playing or praising	Skills that the therapist/program/helper implements during a session to deliver an element	• No adaptions were made regarding the following factors:<br>o Implementation of group rules<br>o Behavior protocols<br>o Group discussions<br>o Group-dynamic games<br>o Behavioral experiments	Discussion with STARC therapists
Treatment delivery	Delivery format	Cultural preferences and acceptability for different treatment modalities	• Support from mental health services is accepted in Germany but less accepted in home countries. [x]<br>• A group setting is usual in German mental healthcare but unusual in home countries. [y]<br>• The concept of the STARC program and its benefit for the participants might be needed to be introduced more in detail in the first session: training can support the participants to cope with the problems and to improve personal and family situations, but the solution comes from the participants themselves, and the new strategies have to be practiced at home to be effective. [z]<br>• The term “training” might be more accepted than “therapy,” given the high stigmatization of psychotherapy and mental disorders. [aa]<br>• Exercises should be adapted according to the level of education of the group members. [ab]<br>• Sessions might need to be further shortened, as the translation takes time. There was little time to discuss personal experiences with the respective topic. [ac]<br>• The translator should have read the group manually to understand the context of the sessions. [ae]	Discussion with STARC therapists

3. Adaptation: Decisions

Decision-No.	Treatment components / delivery	Content / intervention	Translation / Adaptation	Evidence base<br>e.g., literature review, focus groups, qualitative interview	Quality of evidence<br>Strong<br>Moderate<br>Weak	Suggestions from the research team			State of decision
						Researcher 1 AL	Researcher 2 JL	Researcher 3 NN	made
	Unspecific factor	Psychoeducation about the concept of psychotherapy	Inclusion of psychoeducation about the term "therapy" as a common approach in German healthcare to cope with mental health problems and an additional possibility to solve mental health problems within the family.	Focus groups, o, x	Moderate	The concept of psychotherapeutic treatment might be uncommon in home countries.<br>Mental health problems might be solved primarily within the family in the home countries.<br><br>The term "therapy" might be replaced by the term "training" to reduce stigma. However, the term „therapy" in terms of medical treatment might be a familiar concept and might be related to positive expectations.	Mental health problems are mainly solved within families. However, it might be easier to speak with foreigners as there might be less distrust.	Stigmatization of mental disorders is more pronounced in other cultures compared to Germany. Psychotherapy might lead to deterrence, as mental disorders are stigmatized, the use of "therapy" might be more neutral. Some patients prefer the term "therapy" more than the term "training" as they feel taken seriously with their concerns.	x
	Unspecific factor	Psychoeducation about the group setting	Inclusion of psychoeducation about the concept of group therapy as a common approach in German healthcare.	Feedback of STARC therapists, w,y	Weak	Refugees might be unfamiliar with the concept of a group therapy group. Might be helpful to introduce a group setting as a common approach in Germany which can be helpful to support and learn from each other.	Maybe the concept of psychoeducation is an individual approach that is not backed up by the information refugees gave.<br>Psychoeducation might be less common and popular in refugees.<br>Psychotherapeutic jargon and expressions like "psychotherapy" should be reduced in psychoeducation to improve understanding.	Familiarity with the concept of group treatment might differ depending on culture and sociodemographic background.	x
	Unspecific factor	Psychoeducation about individual vs. community perspectives to cope with problems	Further inclusion of psychoeducation about individual vs. community perspectives seems important. Inclusion of psychoeducation about the approach of the STARC program to learn to cope with problems in a group setting, as well as the "individual" approach to equip individuals with new strategies to better cope with emotions.	Discussion with STARC therapists, z	Weak	Cultural differences seem important to be discussed at the beginning of the program as this approach might be uncommon in home countries.	Seems important	Agree.	x
	Specific factor	Psychoeducation about substance use	Inclusion of a discussion of different societal norms for substance use, different availability, and acceptance in Germany and the home country.<br>Inclusion of a discussion of different commonly used substances, e.g., marihuana, alcohol, and prescription drugs	Focus groups, m	Moderate	Discussion of the different handling of substance use might be important to better understand the development of substance use disorder.	The inclusion of a discussion of different societal norms is helpful to find the own position.<br>Provision of marihuana, alcohol and prescription drugs included as examples, which are the most often used types of drugs in Syria.	Agree.	x
	Specific factor	Psychoeducation about substance use disorders	Inclusion of psychoeducation that addiction is a recognized disorder that can be treated.<br>Inclusion of psychoeducation about addiction: Normalization of substance use problems as a coping strategy to deal with	Focus groups, a,b,k,l,m,n,p,r,p, q, r	Moderate	Discussion of different societal norms for substance use seems important to enhance reflection upon reasons for substance use. Normalization of substance use problems seems important to increase perceived acceptance and	The inclusion of substance use as medicine might be helpful.<br>The inclusion of the different social norms can be helpful to find the own position.	All helpful.<br>Individual perspectives/causes for substance use disorders might be difficult to be followed, as usual, the community has the responsibility.	x

Decision-No.	Treatment components / delivery	Content / intervention	Translation / Adaptation	Evidence base<br>e.g., literature review, focus groups, qualitative interview	Quality of evidence<br>Strong Moderate Weak	Suggestions from the research team			State of decision
						Researcher 1 AL	Researcher 2 JL	Researcher 3 NN	made
			stressful experiences.<br>Destigmatization of substance use problems by stressing that substance use is understandable (but harmful) in the face of multiple refugee-specific stressors.<br>Inclusion of the concept of substance abuse as medicine to help to escape from negative thoughts and feelings related to war/trauma.<br>Inclusion of a discussion of risk factors for substance use extended by including: escaping from negative memories, feelings, and traumatic experiences, worries about family members in the home country, not being accepted by the host country, wish of social integration, being in opposition of the IS.<br>Inclusion of a discussion of protective factors: Societal and family norms, religion, social support.			to reduce perceived failure.<br>Connection to stressors/trauma seems important as this explanation might be relieving and may enhance self-acceptance.<br>The concept of substance abuse as medicine to help to escape from negative feelings is a common concept of refugees which seems important to be included.<br>Inclusion of a discussion of the special burden in the homeland, during the escape and in Germany might be helpful.		Joint substance use consumption often serves the need for community and social contact and seems to be an important maintaining factor.	
	Specific factor	Group dynamic game	Introduction of a group dynamic game using a ball was included for both genders, the usage of a ball of wool is only used for female groups.	Discussion with STARC therapists, u	Weak	The usage of a ball of wool was perceived as inappropriate by some of the male participants.	Agree.	A neutral game might be preferable.	x
	Specific factor	Relaxation exercises	Inclusion of a discussion of alternatives to relaxation exercises (e.g., physical exercise, singing) to regulate emotions.	Discussion with STARC therapists, ae	Weak	Inclusion of alternatives for relaxation exercises (e.g. physical exercise) if participants find it difficult to practice relaxation.	The use of relaxation exercises might be culture-specific.<br>In some cultures, relaxation is not a positive thing. People might prefer what is good for them.	Might be preferable to ask the group what might be good alternatives. Singing in difficult times is a very familiar concept for many cultures. Participants need to learn already many new things in their new everyday life, so it might good also to refer to familiar things.	x
	Specific factor	Use of a comparison of a crossroad in Germany compared to home countries	When discussing the two different crossroads, an instruction was included to stress that none of the two crossroads can be placed above the other and that both systems function in their own (cultural) context.	Discussion with STARC therapists	Weak	Agree with researcher 3.	Agree.	Participants might get the impression that the traffic system without traffic lights in the home country are seen as less good compared to the German system.	x
	Specific factor	Use of statements of encouragement as an emotion regulation strategy	Inclusion in the session of the advice that therapists should be careful when using religious statements.	Discussion with STARC therapists, s	Weak	For non-religious group members, non-religious proverbs seem more helpful. Participants might be encouraged to create their own statements that seem most appropriate to them.	I think based on the results of the focus group discussions it is important to avoid religious rituals. They are perceived in a very heterogeneous way.	Religion is a sensitive issue as religion often was a reason for flight.	x

Decision-No.	Treatment components / delivery	Content / intervention	Translation / Adaptation	Evidence base<br>e.g., literature review, focus groups, qualitative interview	Quality of evidence<br>Strong<br>Moderate<br>Weak	Suggestions from the research team			State of decision
						Researcher 1 AL	Researcher 2 JL	Researcher 3 NN	made
	Treatment delivery	Length of session	Long sessions were shortened,	Discussion with STARC therapists, ac, ad	Weak	Some of the sessions seem too long and could not be conducted within the estimated time.	Agree.	A break could be good, especially when very stressful topics are discussed.	x
	Treatment delivery	Type of used language and translation	Use of simple language for all content.<br>Inclusion of instruction for the therapists that the manual content should be adapted according to the level of language skills and education of the group members.	Discussion with STARC therapists, ab	Weak	While easy language seems preferable in general, some of the participants with a high level of education might feel not taken seriously if the language is very easy; therapists may adapt their language to the level of education of the group.	I agree.	The therapist of the STARC group should be able to adapt the content, as this is not possible for the translator. If very easy language is used, some might find the language strange. The language should be understandable for everyone.	x
	Treatment delivery	Translation	Inclusion of the instruction for the therapist that the translator should have read the group manual to understand the context of the sessions.<br>Inclusion of the instruction for the therapist that participants might talk about a stressful situation that might be familiar to the translator and self-care is important.<br>Inclusion of instruction for the therapists that it is important to ensure that the translations need to be understood by all participants that may speak different dialects.	Discussion with STARC therapists, ad	Weak	The translator should be familiar with the content of the manual and the related concepts. Key terms should be translated in the same way throughout the sessions.<br>The translator should be informed about talking about possible stressful experiences and personal triggers.	The translator should have read the manual.	It might be more effective for the participants if the therapists could conduct the group in their mother tongue. Language mediation could be there for the co-therapist.<br>The group needs to pay attention to the different dialects of a language, and misunderstandings have to be discussed as they are likely.<br>The translator should be treated as part of the group.	

Supplement 3.

Sessions of the Adapted STARC-SUD Program

Session	Content
Session 1: Getting to know each other and introduction	Introduction to the program<br>Stressful feelings and the function of addictive substances to cope with such feelings<br>Psychoeducation about the concept of Western psychotherapy that uses individual strategies to improve mental health<br>Psychoeducation about group settings as a common intervention approach in Western cultures to support and learn from each other
Session 2: Perceiving and understanding emotions	Identification of different emotions and their functions<br>Recognizing different intensities of emotions<br>Relationships between specific emotions and substance use
Session 3: Connections between thoughts, body, and feelings	Relationships between thoughts, body, emotions, and substance use<br>Relationships between rumination thoughts and substance use, the importance of substance use in vicious circles of unpleasant feelings, rumination, and consumption<br>Body-based relaxation strategies (e.g., breath exercise)
Session 4: Distressing emotions and substance use	Relationships between intense emotions and substance use.<br>Psychoeducation about the concept of addiction as a recognized treatable mental disorder<br>Psychoeducation about different societal norms for substance use, types of commonly used substances, and their availability and acceptance in the host and the home countries<br>Refugee-specific risk and protective factors of SUD<br>Short- and long-term consequences of substance use<br>Body-based emotion regulation strategies (e.g., Progressive Muscle Relaxation)
Session 5: Emotional warning system: traffic light model	Learning how to use a traffic light model as a warning system for emotions and craving<br>Identifying temptation situations for substance use<br>Body-based emotion regulation strategies (e.g., sports)
Session 6: Thoughts	Relationships between thoughts, emotions, and substance use. Strategies to interrupt negative thoughts and rumination (e.g., breathing exercise)<br>Cognitive emotion regulation approaches (e.g., the shift of attention)
Session 7: Trigger	Personal triggers of intense emotions and substance use<br>Personal temptation situations and the related emotions
Session 8: Action	Skills for coping with intense emotions, tension, or craving (e.g., using a stress ball, chili sweets, a cold shower)<br>Body-based relaxation strategies (e.g., Progressive Muscle Relaxation)
Session 9: Dealing with strong emotions	Identification of specific basic emotions

Session	Content
	Differentiating between healthy and unhealthy emotions and regulation strategies<br>Imaginary emotion regulation strategies (e.g., guided tree imaginary as a source of strength)<br>Strategies to cope with basic emotions (e.g., coping with anger, sadness, or fear) instead of substance use
Session 10: Closing ceremony	Review of the learned knowledge and skills<br>Closing with a joint celebration

Notes. SUD = Substance Use Disorder. STARC = Skills-Training of Affect Regulation – A Culture-sensitive Approach.
